# Simulation of Modal Control of Metal Mode-Filtered Vertical-Cavity Surface-Emitting Laser

**DOI:** 10.3390/s24144700

**Published:** 2024-07-19

**Authors:** Jingfei Mu, Yinli Zhou, Chao Chen, Xing Zhang, Jianwei Zhang, Tianjiao Liu, Zhuo Zhang, Yuehui Xu, Gaohui Yuan, Jiye Zhang, Yongqiang Ning, Lijun Wang

**Affiliations:** 1 Key Laboratory of Luminescence Science and Technology, Chinese Academy of Sciences & State Key Laboratory of Luminescence Science and Applications, Changchun Institute of Optics, Fine Mechanics and Physics, Chinese Academy of Sciences, Changchun 130033, China; mujingfei20@mails.ucas.ac.cn (J.M.);; 2 School of Optoelectronics, University of Chinese Academy of Sciences, Beijing 100049, China; 3 Ace Photonics Company Ltd., Changchun 130102, China

**Keywords:** VCSEL, modal control, metal aperture, modal loss, optical gain

## Abstract

In this study, a novel metal-dielectric film mode filter structure that can flexibly regulate the transverse mode inside vertical-cavity surface-emitting lasers (VCSELs) is proposed. The number, volume, and stability of transverse modes inside the VCSEL can be adjusted according to three key parameters—the oxide aperture, the metal aperture, and the distance between the oxide aperture and the metal aperture—to form a flexible window, and a new parameter is defined to describe the mode identification. This study provides a complete simulation theory basis and calculation method, which is of great significance for the optical mode control in VCSELs.

## 1. Introduction

A vertical-cavity surface-emitting laser (VCSEL) is launched perpendicular to the wafer surface, which is conducive to two-dimensional integration and has the advantages of a single longitudinal mode, low threshold current, low divergence, and low power consumption, making it an attractive optical source. Applications such as optical storage, laser printing, and 3D sensing require single transverse mode, high-power VCSEL light sources [1,2,3,4,5]. Conventional oxide-confined VCSELs usually operate in single mode (SM), with an oxide aperture smaller than 4 μm, which causes increased series resistances and low out power [6,7].

Numerous methods for achieving single-mode output in VCSELs have been documented. These include surface relief VCSELs [8,9], photonic crystal VCSELs [10], impurity-induced disordered VCSELs [11,12], anti-waveguide VCSELs [13], long cavity VCSELs [14], and high-contrast grating VCSELs [15]. Another effective method to achieve a high-power SM-VCSEL is to use a metal aperture for mode filtering [16,17,18]. The analysis in these articles on how metal apertures affect different transverse modes in VCSELs is not exhaustive. In previous studies, we presented a simple modeling analysis of a metal mode-filtered vertical-cavity surface-emitting laser (MMF-VCSEL) [19]. However, there is a lack of detailed analysis of the single-mode characteristics of the MMF-VCSEL and key parameters such as P-DBR, metal aperture size, and oxide aperture size. And there have been no related reports published.

In this paper, a finite element simulation model for a MMF-VCSEL was developed. First, the effects on modal control of variations in the position of the metal layer and the metal apertures and oxide apertures on the fundamental mode optical spot of the MMF-VCSEL were simulated. Then the modal loss and optical confinement factor variations with different metal apertures and metal layer position were systematically calculated. Finally, the optical gain was defined, to describe the modal discrimination and provide alternative computational schemes and parameter ranges to achieve optimal device performance according to specific requirements.

## 2. Structure of MMF-VCSEL

**Figure 1:**
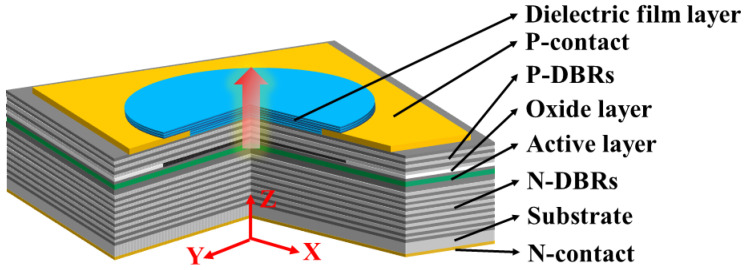
Structure of MMF-VCSEL.

The structure of the MMF-VCSEL is shown in Figure 1. N-DBR is composed of 34 pairs of Al_0.12_Ga_0.88_As/Al_0.9_Ga_0.1_As. P-DBR is composed of Al_0.12_Ga_0.88_As/Al_0.9_Ga_0.1_As, and the number of P-DBRs varies from 4 pairs to 20 pairs. The graphical metal layer is located between the top seven pairs of dielectric films and the P-DBR. The dielectric films are composed of SiO_2_/Nb_2_O_5_. The 30 nm thick oxide layer consists of Al_0.98_Ga_0.02_As, which oxidizes into (Al_x_Ga_1-x_)_2_O_3_.

## 3. Results and Discussion

**Figure 2:**
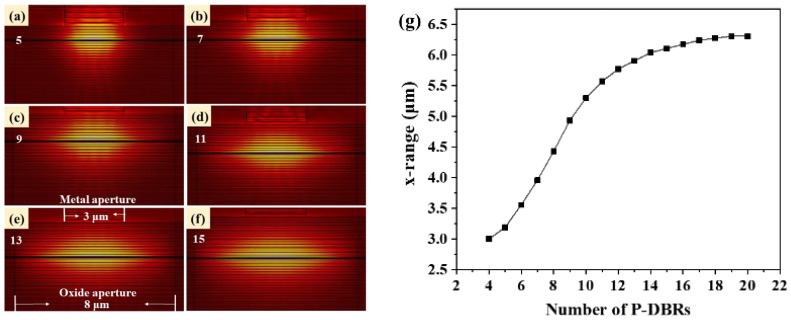
The mode LP_01_ distribution of MMF-VCSEL at (**a**) 5, (**b**) 7, (**c**) 9, (**d**) 11, (**e**) 13, and (**f**) 15 pairs of P-DBRs. (**g**) The x range at which the intensity of the mode LP_01_ optical field decays to one-half as a function of number of P-DBRs.

The performance of the mode control in the MMF-VCSEL is affected by the number of P-DBR pairs. To explore this effect in detail, we have established a finite element simulation model of the MMF-VCSEL using COMSOL (V 4.1) software and carried out detailed calculations. Using Maxwell’s equations and considering the boundary conditions at the core-cladding interface allows for the identification of the waveguide’s supportable transverse electromagnetic modes. Each mode is characterized by its propagation constant and field distribution across the transverse plane [20]. Figure 2 shows the fundamental LP_01_ mode distribution of the MMF-VCSEL at different numbers of P-DBRs, with a 3 μm metal aperture and 8 μm oxide aperture. The optical spot of the LP_01_ mode expands with the increase in P-DBR number and almost distributes across the whole oxide aperture when it reaches 15 pairs, indicating that the limitation of the metal aperture on the transverse optical field is weak. When the number of P-DBRs is small, the metal aperture shows great optical field-limiting performance. The intensity of the LP_01_ mode optical field decays from the center to both sides. When it decayed to one-half of the maximum intensity, we took the range in the x direction as the vertical axis in Figure 2g. The change in the fundamental LP_01_ mode spot size is subtle for more than 15 pairs of P-DBRs.

**Figure 3:**
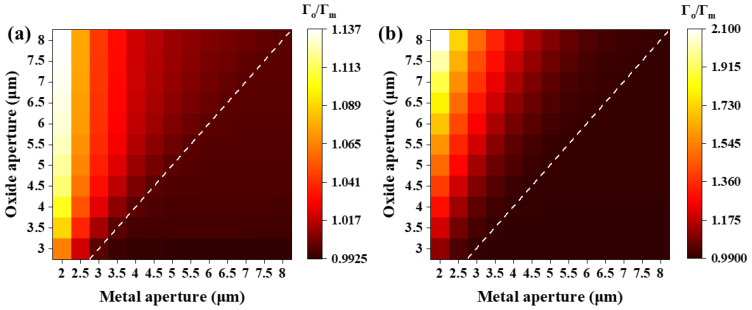
The ratio of the transverse oxide optical confinement factor to metal optical confinement factor of the LP_01_ mode with varying oxide aperture and metal aperture. The numbers of P-DBRs are (**a**) 7 and (**b**) 15. Dashed lines represent points with ratio of 1.

Regarding the MMF-VCSEL as an equivalent cylindrical waveguide with the metal-dielectric film interface as the boundary, to clearly represent the distribution ratio of the optical field in the core and the cladding, we have defined the transverse metal optical limiting factor, which can be expressed as [6]:

(1) $$\Gamma_{m,\;\mathrm{mn}}=\int_{0}^{z}\int_{0}^{r_{m}}\int_{0}^{2\pi }\left|E_{\mathrm{mn}}^{2}\right|d\emptyset \mathrm{drdz}/\int_{0}^{z}\int_{0}^{\infty }\int_{0}^{2\pi }\left|E_{\mathrm{mn}}^{2}\right|d\emptyset \mathrm{drdz}\;$$

where z is the cavity length, $r_{m}$ is the radius of the metal aperture,$\;E_{\mathrm{mn}}$ is the electric field of mode ${\mathrm{LP}}_{\mathrm{mn}}$ of the MMF-VCSEL. Correspondingly, the transverse oxide optical limiting factor can be defined as:

(2) $$\Gamma_{o,\;\mathrm{mn}}=\int_{0}^{z}\int_{0}^{r_{o}}\int_{0}^{2\pi }\left|E_{mn}^{2}\right|d\emptyset drdz/\int_{0}^{z}\int_{0}^{\infty }\int_{0}^{2\pi }\left|E_{mn}^{2}\right|d\emptyset drdz$$

where $r_{o}$ is the radius of the oxide aperture. The variations in $\Gamma_{o}$/$\Gamma_{m}$ of the fundamental LP_01_ mode with different oxide apertures and metal apertures are illustrated in Figure 3.

When the metal aperture is smaller than the oxide aperture, as the metal aperture decreases and the oxide aperture increases, the ratio increases. This variation is relatively small for 7 pairs of P-DBRs, as shown in Figure 3a, while it becomes significant for 15 pairs of P-DBRs, as illustrated in Figure 3b. It is caused by the strong confinement of the transverse mode by the metal aperture with 7 pairs of P-DBRs, while the opposite is observed with 15 pairs, as shown in Figure 2. The strong confinement from the metal layer results in the fundamental mode spot size changing with the metal aperture. The majority of the optical field remains within the metal aperture, so the ratio does not strongly depend on variations in the integration region, as shown in Equations (1) and (2). The weak confinement of the metal aperture leads to the opposite outcome. When the metal aperture is greater than the oxide aperture, the ratio undergoes extremely small changes, and the minimum value exceeds 0.99. This indicates that the fundamental mode optical field is almost entirely confined within the oxide aperture, even for seven pairs of P-DBRs. Therefore, the restriction of the optical field modes by the metal layer is not only related to the distance between the metal layer and the oxide layer. It is also subject to competition between the size of the metal aperture and the oxide aperture. The side with the smaller aperture wins the right to limit the optical field.

Reaching the threshold is a necessary condition for VCSEL modes excitation. This threshold condition is met when the modal gain equals the modal loss in the cavity. The threshold gain can be expressed as [21,22,23]:

(3) $${\Gamma g}_{\mathrm{th}}=\alpha_{\mathrm{in}}+\alpha_{\mathrm{mirr}}$$

where [23]

(4) $$\Gamma =\int_{\mathrm{active}}\left|E_{\mathrm{mn}}^{2}\right|\mathrm{dV}/\int_{\mathrm{cavity}}\left|E_{\mathrm{mn}}^{2}\right|\mathrm{dV}$$

Is the optical confinement factor, which represents the fraction of the optical field that is confined within the active region of the VCSEL. ${\Gamma g}_{\mathrm{th}}$ is the threshold gain, representing the gain needed to compensate for the loss in the cavity. $\alpha_{\mathrm{in}}$ is the intrinsic loss, and $\alpha_{\mathrm{mirr}}$ is the mirror loss expressed as [21,22]:

(5) $$\alpha_{\mathrm{mirr}}=\frac{1}{L_{\mathrm{eff}}}\ln\frac{1}{\sqrt{R_{t}R_{b}}}$$

where $L_{\mathrm{eff}}$ is the effective cavity length. We assume that the reflection of the P-DBR and N-DBR separately effectively originates from a hard mirror with an identical peak reflectivity placed at a distance. The distance between the two hard mirrors represents the $L_{\mathrm{eff}}$. $R_{t}$ and $R_{b}$ denote the reflectivity of the top mirror and the bottom mirror. The optimization of the thickness of the metal layer, taking into account the light absorption of the material, has been discussed in our previous work [19], and the thickness of the metal layer is determined to be 900 nm.

**Figure 4:**
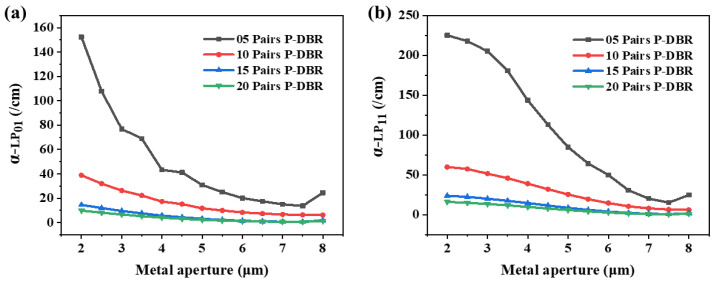
The modal loss of (**a**) LP_01_ and (**b**) LP_11_ at various metal apertures with various numbers of P-DBR pairs. The oxide aperture is 8 μm.

Figure 4 shows the variation in modal loss of LP_01_ and LP_11_. The modal loss of LP_11_ is greater than that of LP_01_ for all metal apertures and numbers of P-DBRs. It is due to the fact that the optical spot of the mode LP_11_ is distributed closer to the edge of the aperture. The modal loss of both modes decreases as the metal aperture increases, and this change is more pronounced with fewer P-DBRs. However, the trends of the two modes are different, which is more evident with five pairs of P-DBRs. This may be due to the different sensitivities of the distributions of the two modes to the size of the metal aperture. This is what makes it possible to set appropriate structural parameters to achieve a large difference in the two modal losses, which will be beneficial for the modal discrimination of the MMF-VCSEL.

**Figure 5:**
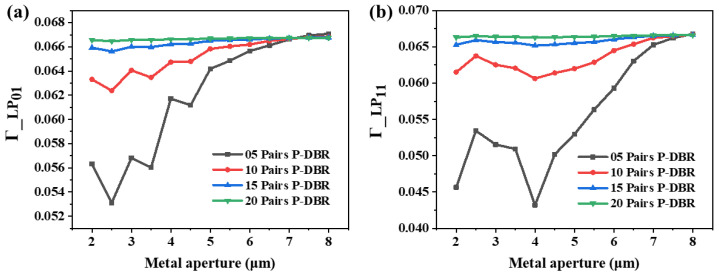
Optical confinement factors of (**a**) LP_01_ and (**b**) LP_11_ at various metal apertures with various numbers of P-DBR pairs. The oxide aperture is 8 μm.

Figure 5 shows the variation in the optical confinement factors of the LP_01_ and LP_11_ modes. The Γ of mode LP_01_ is slightly larger than that of mode LP_11_, except at a 2.5 μm metal aperture, and the small difference is related to the large oxide aperture of 8 μm. The Γ of the two modes change in a tortuous and different process with the increase in metal aperture. This is also due to the different sensitivities of optical field distributions of the two modes in the active region to the sizes of the metal apertures. Because their respective trends are the same with different P-DBR numbers, as shown in Figure 5a,b, the Γ of the two modes both have a strong dependence on the metal aperture. Especially at a metal aperture of 4 μm, the LP_01_ mode obtains a large optical confinement factor, while mode LP_11_ reaches a minimum value at the same number of P-DBRs. This is caused by the introduction of the metal layer inside the cavity, leading to transverse resonance [18]. This transverse resonance is related to the distance between the metal interface and the oxidation interface. The resonances of the LP_01_ mode and the LP_11_ mode differ due to their spatial distribution and wavelength differences. Additionally, the result of this transverse resonance directly affects the optical field distribution in the active region, leading to changes in the confinement factor. When the metal aperture is 4 μm, the stable transverse resonance of the LP_11_ mode is disrupted, significantly affecting its intracavity optical field distribution, thereby reducing the Γ of the mode LP_11_ to a minimum.

**Figure 6:**
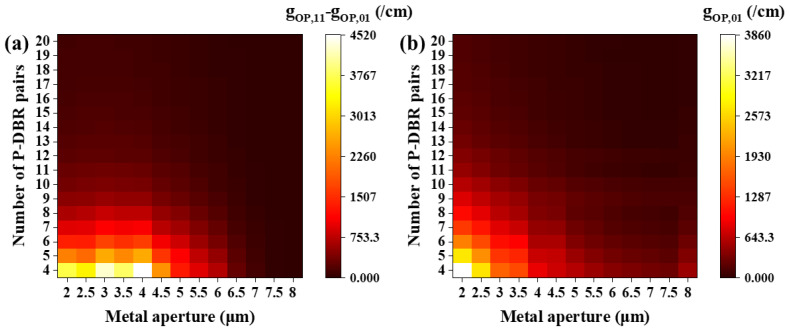
(**a**) The difference in optical gain of the two lowest-order modes. (**b**) The optical gain of the LP_01_ mode at various metal apertures with various numbers of P-DBR pairs. The oxide aperture is 8 μm.

In Equation (3), the internal loss $\alpha_{\mathrm{in}}$ is mainly affected by the doping concentration of the VCSEL epitaxial material, which is a constant value for determining the structure. If the internal loss is ignored, a new variable called optical gain ($g_{\mathrm{op}}$) can be defined, whose physical significance is to characterize the change in threshold gain of different transverse modes $LP_{mn}$ in the VCSEL due to optical scattering by the structure. The expression of $g_{\mathrm{op}}$ is written as:

(6) $$g_{\mathrm{op},\mathrm{mn}}=\alpha_{\mathrm{mn}}/\Gamma_{\mathrm{mn}}$$

where $\alpha_{\mathrm{mn}}$ is the modal loss of the $LP_{mn}$ mode and $\Gamma_{\mathrm{mn}}$ is the optical confinement factor. The larger $g_{\mathrm{op},\mathrm{mn}}$ is, the more difficult it is for the mode $\;LP_{mn}$ to lase, because its threshold gain is also larger. Figure 6a shows the variation in the optical gain difference between the LP_11_ mode and the LP_01_ mode. This difference increases as the number of P-DBRs decreases at all metal apertures, and it obtains a maximum value at a metal aperture of 4 μm. This indicates that the closer the metal layer is to the oxide layer, the stronger the mode discrimination of the MMF-VCSEL, which is benefit for single-mode performance. The maximum difference is related to the large mode loss difference and optical confinement factor difference obtained by the MMF-VCSEL at a 4 μm metal aperture, as shown in Figure 4 and Figure 5.

A large optical gain difference is expected, and the optical gain of the LP_01_ mode also needs to be considered. A large $g_{\mathrm{op},01}$ is unacceptable and not conducive to the slope efficiency of the MMF-VCSEL. Figure 6b shows the change in $g_{\mathrm{op},01}$ with varying metal apertures and P-DBRs. As the number of P-DBRs and the size of the metal aperture decrease, the $g_{\mathrm{op},01}$ increases. Therefore, through the above calculations, considering the values of the optical gain difference and $g_{\mathrm{op},01}$ simultaneously, a balance in terms of the mode discrimination and slope efficiency of the MMF-VCSEL can be found, and finally the optimal structural parameters will be determined.

## 4. Conclusions

In this study, the strong regulatory effect of metal-dielectric film mode filters on the transverse optical mode in VCSELs is discussed. The transverse mode in the MMF-VCSEL can be flexibly modulated, because the metal-dielectric thin film structure has different scattering effects on different transverse modes. When the metal aperture is smaller than the oxide aperture, the optical scattering effect is enhanced with the decrease in the distance between the metal aperture and the oxide aperture, resulting in an increase in mode discrimination and mode loss at the same time. By selecting appropriate metal aperture and oxidation aperture values, the VCSEL can maintain good single-mode stability while having low threshold characteristics. When the metal aperture is larger than the oxide aperture, the optical mode in the VCSEL is mainly controlled by the oxide aperture. By defining a new variable, optical gain, a selectable computational scheme balancing between the single-mode stability and slope efficiency of the MMF-VCSEL is provided.

## Data Availability

The data presented in this study are available on request from the corresponding author.
